# "The Hospital" Nursing Mirror

**Published:** 1898-11-05

**Authors:** 


### "The Hospital" Nursing Mirror.

BEING THE NURSING SECTION OF "THE HOSPITAL."

[Contributions for this Section of "THE HOSPITAL" should be addressed to the Editor, THE HOSPITAL, 28 & 29, Southampton Street, Strand, London, W.C., and should have the word "Nursing" plainly written in left-hand top corner of the envelope.]

### News from the Nursing World.

### THE NEW NURSES' HOME AT SUNDERLAND.

WE have received further particulars of the new home for the nurses of the Sunderland Nursing Institute opened on the 23rd ult. by the Marchioness of Londonderry. It has been named Victoria House, is situated in Murton Street, and has been carefully re-arranged and adapted for its new purpose. It contains eight bedrooms, a private sitting-room for the superintendent and one for the nurses. The dining-room is directly over the kitchen, and there is a lift between the two. One large room has been completely fitted up for the nursing appliances and stores for use in the district. The home, which was opened by the president, Lady Londonderry, is quite distinct, however, from her ladyship's work at Stockton, with which it has sometimes been confused. There is no debt upon the building, as a sufficient sum—£1,714—was allotted to the association from the Sunderland Diamond Jubilee Fund. It is hoped that it will be furnished without encroaching on the general resources. There has been a district nursing association in the town for some years, but in January last the work was handed over to the Queen's nurses, who, until the new home was opened, had been living in lodgings. The staff at present consists of a superintendent and three nurses, but the work is already beyond their scope.

### NURSE PECHA'S DEATH.

NURSE PECHA died on the 30th ult. of the plague, which she contracted attending on Barisch. The exact place of her burial will be kept secret, as, on account of the prevalent sympathy for her untoward fate, her grave would be made an object of pilgrimage. Nurse Pecha was only 22 years of age; she was the daughter of a railway labourer in Bohemia, and the youngest of nine children. She was sent to service as early as possible, and subsequently became chambermaid at Carlsbad. There she nursed an invalid Irish gentleman so well that he took her to Ireland, whence she went to Vienna in order to be properly qualified as nurse. She was attached to the hospital as reserve nurse, and was, therefore, only called upon to attend Barisch because the regular staff were overworked at the time.

### BRISTOL GENERAL HOSPITAL.

AN enjoyable "At Home" was given at the Bristol General Hospital on the occasion of the prizegiving to the nurses on Friday, October 28th, 1898. Mr. Joseph Storrs Fry, chairman of the committee, took the chair, and there were present several members of the committee and of the medical staff. In general nursing, for those of three years' training, Nurses Swain and Bottomley took first and second prizes. In anatomy, Nurse Swain the second (the first not being awarded); and in physiology, Nurses A. Bale and Swain received first and second prizes respectively. For nurses of two years' training, Nurses E. Bale and Wren took first and second prizes respectively in general nursing; Nurses Flamstead and Richards first and second in anatomy; and Nurses E. Bale and Edwards in physiology; whilst Nurses Watson and Hamblin took first and second prizes in general proficiency.

### WORKHOUSE NURSES AND HOSPITAL CERTIFICATES.

A MATTER of some importance was decided, and fortunately was decided rightly, at the last quarterly meeting of the governors of the Norfolk and Norwich Hospital. It appears that the nurses from the workhouse are allowed to attend the lectures given by the medical staff to the nurses of the hospital, which is a very proper and useful arrangement; but when it is urged that the workhouse nurses, on completing their course, should receive certificates the affair assumes an altogether different aspect. It must never be forgotten that the training of a nurse is to be obtained only by practical experience, and that lectures, useful as they are in enabling a nurse to understand the reason of much that she does, do not in themselves in the slightest degree qualify anyone for the work of nursing. The certificate must be given by those who have watched the nurses in the wards, and may perfectly well be made to include a statement that certain lectures have been attended; but where these lectures are attended, and by whom they are given, is purely a matter of arrangement between the authorities. We think, however, that any workhouse infirmary superintendent, who proposes to send her probationers outside for their lectures, would do well to ascertain beforehand whether these lectures will be recognised by the Local Government Board as qualifying for the post of superintendent nurse, because, so far as the wording of the order of the Board by which this grade was instituted goes, there is no reference to any such arrangement. Unless the point is made clear it might so happen that a considerable injustice might be done, and that at the end of their term the probationers might find their certificates unrecognised.

### THE WHITE-HANDED NURSE.

THE beauty of a well-kept hand appeals to all, and perhaps most of all to the genuine nurse, who has so often to sacrifice the whiteness and softness of her own to the exigencies of antiseptics. The fact that there are women calling themselves nurses who, earning a livelihood by attending the sick, yet value the appearance of their hands more than their patients' comfort, will therefore occasion those who are nurses indeed an amusing surprise. Nevertheless, it is true. One such person, who lately sued her patient for fees, left the house because she was asked to let down the blinds, "because her hands was tender." The stamp of woman, and the excuse she gave for not doing as she was asked, is too incongruous not to be ridiculous, and it is a pity that this kind of nurse could not, for the safety of the public, be branded "Mrs. Gamp."

### WARRINGTON NURSES' HOME.

The Warrington District Nursing Association have the satisfaction of feeling that the association has supplied a real want in the neighbourhood. The home in Arpley Street has been comfortably furnished for three nurses and a maid-servant. The nurses have been fully occupied, and the inspector of the Queen Victoria's Jubilee Institute for Nurses (to which institute the home is affiliated) issues a favourable report. The financial support afforded by the townsfolk has also been generous.

### THE ROYAL NATIONAL PENSION FUND FOR NURSES.

We are pleased to announce that the Junius S. Morgan Benevolent Fund, in connection with the Pension Fund, has found a kind friend as far away as Natal, in the person of Nurse Mary Roberson, of Durban, who has just forwarded a sum of £2 10s. 6d. to the fund. This has been collected amongst friends and patients, and shows a most kind thought for her fellow-workers less fortunately placed than herself.

### CHELTENHAM DISTRICT NURSING ASSOCIATION.

Dr. T. E. Wilson presided over the annual meeting of the Cheltenham District Nursing Association, and Miss Goslett read a paper on the value of trained nursing to the poor. Thanks to the Jubilee gift of £1,025 in 1887, the nursing association was put on a much sounder financial basis, whilst another of £1,687 last year enabled the committee to add considerably to the endowment fund, and to buy the house adjoining the Victoria Home. The training of the probationers, of whom there were three, reflects great credit on the lady superintendent. Miss Peter, inspector of the Queen Victoria's Jubilee Institute for Nurses, asked for seven last January, but only three were available. The staff consists of four general and three maternity nurses, and the average number of visits per diem is 50.

### FLANNEL SHIRT CLUB.

Last year the Flannel Shirt Club was founded, under the presidency of the Countess of Strafford, and the first report shows a very satisfactory growth. The object is, as we noted at the time it was founded, to provide working men convalescents with warm shirts upon leaving the hospitals. Full particulars may be obtained from the secretary, Miss Lamport. The names of Lady Muriel Boyle, Miss Gethen, and Miss Phillipa Hicks are associated with the committee, and about 700 shirts were distributed during the year. We are glad to note that flannelette is not allowed, for useful as the latter material is, it is vastly inferior to flannel for the purpose mentioned.

### NURSING AT READING.

The first report of the Queen Victoria Institute at Reading is pleasant reading. There have been many kind friends to take a personal interest in it, and some have been very generous. The endowment fund has reached the respectable total of £3,027, and the maintenance fund £359, whilst Mr. M. J. Sutton has kindly discharged the furnishing account, amounting to £250. At the annual meeting the Mayor presided, and a large and representative company were present. The chairman, with reference to establishing a provident side to the work, remarked that it would take 400 members paying 2s. 6d. a-year each to pay for a nurse, and they would run a certain amount of risk in pledging themselves (as they would have to do) to provide these 400 subscribers with a nurse whenever any one of them thought he ought to have one.

### A CORRECTION.

A curious mistake crept into a paper on "District Nursing Organisations" read at the Congress of Women Workers by Lady Laura Ridding. Lady Laura stated that "a fully-trained nurse, whose minimum qualification is one year's general hospital or infirmary training, where there is a resident medical staff, followed by six months' district training, can earn a salary of £60 to £90 per annum," while the salaries of "village nurses" vary "from £35 to £50 per annum." Miss Amy Hughes explained, in the course of the discussion which followed the reading of the paper, that "£60 to £90" was not for salary only, but included maintenance; but in her reply, Lady Laura still appeared to cling to the belief—may it soon be realised!—that a fully-trained district nurse earns this noble income. As a matter of fact, of course, the confusion is between salary, as distinct from maintenance, and salary and maintenance taken together as the total cost of a fully-trained district nurse. The standard adhered to by the authorities of the Q.V.J.I.N., as the proper average for keep and salary of a Queen's nurse, is from £60 to £90. The actual salary, apart from maintenance of a Queen's nurse, averages, we believe about £35 per annum.

### NURSES' HOME FOR SELLY OAK WORKHOUSE.

Mr. J. Walter, chairman of the House Committee of the King's Norton Board of Guardians, formally opened a new home for the night nurses of the Selly Oak Infirmary. The name of this new acquisition is Oaklands, and it is a large residence standing on a site recently purchased by the Guardians. As there is sufficient accommodation for the day nurses at the infirmary, the home has been allotted to the night nurses, who needed it badly.

### SHORT ITEMS.

An anonymous donor has given £50 to the Loughborough Nursing Association. The nurse has been obliged to resign through over-work. There is urgent need of a second nurse.—It appears that Nurse Pecha was most probably infected with the plague by breathing the germs coughed up by Barisch, who first caught the disease through the respiratory organs whilst feeding the animals under experiment. The nursing staff in the infectious hospital at Vienna have been provided with "bacteria filters," to guard against their inhaling the infection. Nurse Pecha and others exposed to infection have been inoculated with serum.—The result of the bazaar held in July at Inveraray was very satisfactory. The Duchess of Argyll receives £200 for the Argyllshire Nursing Association, and the Inveraray branch of the Young Women's Christian Association £20. The Nursing Association have recently appointed a nurse for the Craignish district.—Winslow is fortunate in obtaining the services of a "Queen's" nurse. Three ladies—Mrs. Lambton, Mrs. Greaves, and Mrs. McCorquodale—have interested themselves in the matter.—Several ladies and gentlemen, students at the Royal Orthopædic Hospital, Oxford Street, gave a second concert one day last week to the patients and nurses.

### Nursing in Diseases of the Throat, Nose, and Ear.

By MACLEOD YEARSLEY, F.R.C.S.Eng., Assistant Surgeon to the Royal Ear Hospital, Surgeon-in-Charge of the Department for Diseases of the Throat, Nose, and Ear, the Farringdon General Dispensary, Hon. Surgeon for Diseases of the Throat and Ear, the Governesses' Home, &c.

### V.—OPERATIONS ON THE EAR.

In operations on the ear it may again be said that the general preparation of the patient will not differ from that for any other surgical procedure. The local preparation of the ear is, however, of great importance, and upon its proper purification depends to a considerable extent a good result. The ear will stand very strong antiseptics, and this fact is made use of in purifying it for operation. There are several methods used, and different surgeons prefer different antiseptics; there is no need, however, to describe them all, and the method in vogue at the Royal Ear Hospital will, therefore, be the only one detailed, and is as follows: Having syringed the ear with a warm antiseptic to clear out all discharge, the auricle and surrounding part is first carefully washed and purified with 1 in 40 carbolic, care being especially taken to thoroughly clean the nooks and crannies made by the foldings of the cartilage. The ear is then syringed with warm 1 in 40 carbolic and plugged with a strip of gauze wrung out in the same solution; a pad of this material is then placed in the concha, some absorbent wool and a bandage keeping the whole in place. At the operation a piece of mackintosh sheeting is placed beneath the head and over the shoulders, and this is covered by a towel wrung out in 1 in 20 carbolic, part of the towel (or a separate one) being wrapped around the head, covering the hair. The surgeon removes the gauze from the ear and proceeds to purify it further by first syringing it with warm 1 in 20 carbolic and finally mopping it out with "strong mixture."* This final purification by the surgeon is, of course, done when the patient is anæsthetised. The operation is then proceeded with.

In the first cleansing of the ear one of the numerous ætherial liquid soaps is very useful, as it removes all greasy material without the preliminary application of ether or ammonia. In operations where a portion of the head requires shaving it is well to do this first, as the shaven part and the ear can be then purified at one and the same time.

The above method applies more especially to operations done under general anæsthesia. In those smaller manipulations done under cocain in the out-patient room the surgeon usually purifies the ear at once, a proceeding which is too often only done in a perfunctory manner, especially in those ear departments in general hospitals which are placed in the care of young general surgeons who are playing at specialism.

As regards the preparation of snares, what has been said in referring to operations upon the nose applies also here.

The smaller operations, often done in the out-patient room, are the following: (1) Removal of polypi and granulations; (2) paracentesis of the drum membrane; (3) opening of furuncles. These may all be done under cocain, eucain, or general anæsthesia by gas, chloroform, or ether.

Removal of Polypi or Granulations.—The instruments required are: Specula, probes, wool-armed probes, snare, curette. The head should be gently but firmly steadied by the nurse. Snares vary in pattern; the best is that designed by Mr. Cresswell Baker. Curettes are also of various patterns, that of Burkhardt-Merian is the most useful one. After the curetting, the ear is plugged with gauze, wrung out in 1 in 40 carbolic.

Paracentesis of the Drum Membrane.—The instruments required for this simple but valuable operation are specula, wool-armed probes, and paracentesis knife. The paracentesis knife (or myringotome) varies in pattern. A spear-shaped instrument or a small pointed knife may be used. A fine tenotomy knife will answer the purpose. The ear is plugged, as usual, after the operation. The nurse will be again required to steady the head in this and the next operation.

Opening Furuncles.—It is to be doubted whether any ear disease causes more actual distress and suffering than boils in the external meatus. Relief on incision is prompt, and may be afforded under cocain, eucain, or gas. The instruments required are speculæ and furuncle knife. This opportunity may be taken to mention the useful operating speculum of Pritchard. This is merely an ordinary speculum, with a part of one side of the narrow end removed. The meatus must be purified first, but, owing to its exquisite tenderness, this should be done under cocain anæsthesia, and should consist of careful mopping with a solution of perchloride of mercury of the strength of 1 in 1,000. If the boil is opened under cocain or eucain, the head requires very careful and firm steadying, and precautions must be taken to prevent the patient knocking up or seizing the operator's hand.

Of the more serious operations, the chief ones to be described are: Excision of the ossicles, the removal of exostoses, mastoid operations.

Excision of the Ossicles.—The instruments required are: Syringe, speculæ, special knife, Delstanche's extractor or Sexton's pincette, incus hook, probes, curette, small sponges on forceps (small pieces of sponge on ordinary Spencer Wells' forceps may be used, or they may be placed in special sponge holders), or small cotton-wool swabs, and ear forceps. The hæmorrhage in this operation is somewhat free; it can, however, be sufficiently controlled by a preliminary instillation of cocain, pressure by sponges, &c., or by peroxide of hydrogen. When the operation is finished the nurse should have ready the syringe and some hot antiseptic for syringing. The ear is finally dressed as already described. It is as well to mention that in all operations the nurse should see that everything removed is kept for the surgeon's inspection.

Removal of Exostoses.—There are several methods of operating for exostoses of the external meatus. Some surgeons use the drill, others prefer the mallet and chisel. At times it is necessary to detach the auricle and turn it forward. The preparation of instruments therefore depends upon the operation to be done. When the chisel and mallet are used, the following will be necessary: Speculæ, probes, small chisels,* mallet, forceps, small sponges or cotton wool, probes, syringe. If it is necessary to turn forward the auricle, a scalpel, Spencer Wells' forceps, needles, and sutures will be required in addition. Gauze strips for plugging, hot antiseptic solution, and peroxide of hydrogen for hæmorrhage should also be at hand.

Mastoid Operations.—Some surgeons operate with the trephine, others with the drill or burr, whilst a third group use the chisel and gouge. Personally I prefer the last-named method. Before the operation, the side of the head requires shaving, an area of clear skin with a radius (measured from the middle of the meatus) of about three and a half to four inches being necessary. It is better to shave too much than too little. This area and the ear itself must be carefully purified. When the patient is on the table the head must be surrounded with towels wrung out in 1 in 20 carbolic as already directed. The instruments necessary are: Scalpel,

* Lister's "strong mixture" consists of a solution of 1 in 25 carbolic acid, with 1-500th part of perchloride of mercury.

* The small dentine chisels used by the dentists are most useful.

syringe, Spencer Wells' forceps (at least a dozen pairs), mallet, chisels, gouges,* blunt retractors, curettes, sharp spoons, autrum guide, ligatures, needles and sutures, wool-armed probes or small sponges on forceps or handles, forceps (dissecting and ear). There should be at hand plenty of hot water and antiseptics, cotton wool swabs. Peroxide of hydrogen is often useful; gauze strips, gauze pad, wool and bandage will be wanted. It is not often necessary to use drainage tubing, but some should be ready in case it is required.

In conclusion, one may mention the removal of tumours from the auricle, and the operations for lateral sinus thrombosis and for brain abscess. As regards the first-named, the operation scarcely differs from the removal of tumours elsewhere. In the case of lateral sinus and brain abscess operations, the surgeon will (unless he has already done so) open the mastoid first. If it is a case of thrombosis of the lateral sinus with which he has to deal, there should be plenty of strips of iodoform gauze at hand for immediate use if required. When it is likely that a brain abscess will have to be opened, trephines, &c., must be put out.

* Unless the operator prefers to use the trephine, drill, or burr; but, even then, it is well to have mallet, chisel, and gouge at hand. It is always better to err on the side of too many than too few instruments.

### District Nursing and Charity.

An interesting paper was read before an extraordinary meeting of the Council of the Charity Organisation Society at the theatre of the Royal United Service Institution, by Mrs. Mary Minet, on Monday, October 31st, of which the subject was "Nursing Associations and Charitable Relief." Mrs. Minet defined the ideal of district nursing as that set forth as the object of Queen Victoria's Jubilee Institute for Nurses, namely, "For nursing the sick poor in their own homes free of charge." After questioning whether district nursing could be justified in accordance with the principles of the Charity Organisation Society, she replied that, in her opinion, it could. Separating education and medical treatment from other forms of relief, she asserted that "an ignorant man or a sick man was a spot of taint, and that the safety of the people demanded that the spot be cleansed, and at once, without inquiry into the worth of the individual." Having touched upon the expediency of healing the sick, she proceeded to show the utility of the nurses' work. The sick and those around them are in a state of abnormal receptivity, and the object lessons in the primary laws of hygiene, conduct, cookery, cleanliness which a nurse was able to give instructed the people in things never dreamt of before, and was likely to have a more or less permanent effect. Mrs. Minet then stated the desirability of teaching the people to be provident, and the difficulties attendant thereupon. It was easier in the country, but the obstacles occasioned by the shifting populations of the large towns were enormous; still she hoped that "as nursing comes more and more to be popularly recognised as the necessary complement and handmaid of medical treatment, so we might trust would it come under the influence of the same providence." The speaker conceded that it was almost impossible for a superintendent to realise her ideal, namely, that a nurse should only be sent to those who were unable to provide one for themselves. The temptation to do otherwise came largely from medical men, who, when they took a professional interest in a case, thought more of obtaining a reliable nurse than of the patient's ability to pay. A superintendent was also tempted to take difficult cases for the advantage it afforded her probationers. Not only was unrestricted district nursing an evil in itself, but it was an injustice to the daily or visiting nurse who had her living to make by it. Mrs. Minet suggested that the nursing homes should have a visiting nurse or nurses in connection with them, so that when a doctor sent in a case that could afford to pay such a one should be sent instead of the district nurse. She also thought that it would be advisable for every superintendent to be an *ex officio* member of the Charity Organisation Society, and that she should keep the society informed of the knowledge that they require, and which she is in such an advantageous position to acquire.

The discussion following was instructive, and Mr. Loch's (the secretary of the Charity Organisation Society) statement of the attitude of the Charity Organisation Society to the district nursing associations was met with marked approval, whilst the Chairman's summing-up was excellent. The gist of the whole matter was that nurses must not give relief; they must confine themselves, however hard it may be in individual cases to refrain, to nursing alone. The second point was the desirability of all paying for nurses' attendance in accordance with their power, the most feasible suggestion to attain this result being that recommending the consolidation of nursing agencies. A third point, urged by Mr. Loch, was the instruction of nurses into the methods of the Charity Organisation Society during training if possible, or by the society approaching the nurses, especially the superintendent. One lady remarked that the danger of teaching people to expect more than they paid for, because they had paid a little—the principle of the benefit societies—was more harmful than the giving of charity. Mr. Tennant asked if it would not be possible for the nurses to have forms to fill in as to the circumstances of the patients, and so facilitate the work of the Charity Organisation Society and avoid delay in urgent cases; and Mr. Connelli, as to whether the great friendly societies had taken any steps to provide nurses for their members. To this last query information was volunteered that they did not do so; but occasionally they granted subsidies to the district nursing association, but only to small amounts. Mr. Loch argued that the charity of the nurse ended with the recovery of her patient, but that that of the Charity Organisation Society in worthy cases did not end until he was set upon his feet again.

### Minor Appointments.

The Infirmary, Stockport.—Miss Sybil Fames was elected Charge Nurse of this infirmary on September 5th. She was trained at the Manchester Royal Hospital, and since then she has been on the private staff of the Southport Infirmary, engaged in private work in Canada (where she took locum tenen's duty for the matron of the Children's Hospital at Ottawa), and for five years sister of a private surgical hospital in Manchester.

Oldham Infirmary.—Miss Bertha Kay Collis, who was trained at Bridgewater Infirmary, and who was subsequently staff nurse of the above infirmary, was promoted on October 28th to the post of Sister of the male wards.

Selly Oak Infirmary, Birmingham.—Miss E. A. Butler, who was trained at Preston Royal Infirmary, has been appointed "Home Sister" of the new Home for Night Nurses recently opened.

Tonbridge Union.—On October 12th, Miss Minnie Alder was appointed Assistant Nurse here. She was trained, under the Meath Workhouse Attendants' Association, at the Home for Invalids, Aubert Park, Highbury, N.

Colchester Hospital.—Miss Margory Bridger, who was trained at the Royal United Hospital, Bath, has been appointed Sister here.

### The National Union of Women Workers.

ANNUAL CONFERENCE AT NORWICH.

The annual conference of the National Union of Women Workers, held during the past week in the old cathedral city of Norwich, may with truth be said to have run its course with all success. The meetings were productive of plenty of interesting discussion, and the interchange of valuable and often expert knowledge amongst the members cannot but prove helpful to all who were present. Taking a bird's-eye view, as it were, of the week's doings, it strikes one as a hopeful augury for the future that so many women of all ranks whose names are associated with high and noble work should gather together from all parts of England to consider how to solve some of the greatest problems of our complicated modern social system. All who know Liverpool are aware of the good works with which Mrs. Alfred Booth, the President of the Union for this and for next year, is connected, and the names of the Countess of Leicester, the Countess of Meath, Lady Battersea, Lady Laura Ridding, Mrs. S. A. Barnett, Mrs. Henry Sidgwick, Mrs. Boulnois, Mrs. Burgwin, and a long roll of others too numerous to mention, are familiar in the ears of those who interest themselves in the public doings of English women. Norwich gave the Women Workers a cordial reception. The houses in and around the city were filled with guests, and the members of the Local Conference Committee, of which Lady Leicester was patroness, and Mrs. Sheepshanks president, must indeed have worked hard to get the arrangement machinery into such good order. The Bishop and Mrs. Sheepshanks were "at home" to a number of the delegates on the evening before the Conference opened, a pleasant inaugural gathering, much enjoyed by all who were present. On the Thursday evening, when the chief business of the Conference was over, the Mayor and Mayoress received all the members in the beautiful old building, once a Dominican church, now known as St. Andrew's Hall. Special services at the Cathedral on Friday, which were very largely attended, concluded the week's proceedings. On Friday, by special invitation, a large party of the delegates visited the famous Carrow Works, whence Colman's mustard finds its way to all parts of the world. After hearing much of the horrors of "home work," it was refreshing to see an absolutely model factory, with its kitchen, dispensary, dining rooms, &c., where the people are cared for in every possible way, the late Mr. Colman and all his family taking personal interest in every detail affecting the well-being of those employed in the works.

It would, perhaps, have been hard to find a more attractive old city in which to hold such a meeting as the Women's Conference, associated, too, as it is with the names of more than a few famous women of the past. Norwich was the birthplace of Elizabeth Fry, of Harriet Martineau, of Amelia Opie, it was the home of the Taylors, and has association with many another well-known name, notably the Gurney and Buxton families, which were together represented upon the Local Committee by no less than ten ladies. The family of Colman, too, whose work in Norwich and amongst their own people is well known, were represented by several members. But space fails for further dilatation upon Norwich and its pleasant associations. We must come to the actual work of the Conference.

Women's Views on Current Questions.

The members of the conference mustered in strength at the opening meeting on the morning of Tuesday, the 25th. Mrs. Sheepshanks, on behalf of the Norwich Committee, welcomed the visitors in a brief speech, and then came Mrs. Booth's presidential address, delivered with all the charm of manner which makes her an ideal president, in which she made special mention of the indefatigable labours of Miss Janes, the secretary of the N.U.W.W.

Technical Education.

The first subject upon the programme, "Technical Education for Girls from Elementary and Secondary Schools," was introduced by Mrs. Arthur Francis, a school manager in Chelsea under the London School Board. Mrs. Francis contended for the importance of manual training, "a complete training of the mind through the hand and eye, side by side, and in conjunction with the training of the brain." Other speakers were Miss Sproule, Inspector of Domestic Subjects under the West Riding of Yorkshire County Council, Miss Fanny Calder, and Miss Angwin, after which the discussion drifted decidedly wide of the original subject, and resolved itself into somewhat special pleading on behalf of agriculture as a profession for women, interesting, but hardly in place.

Midwifery and Maternity Nursing in Villages.

The interest of nurses present at the conference naturally centred in the afternoon's meeting, when papers on the "Work of Midwives and Maternity Nurses" were read by Lady Laura Ridding and Sister Katharine Twining, of Plaistow, Lady Battersea presiding. Lady Laura dwelt upon the need for a further supply of nurses in country districts, where ambulance aids, hospitals, and medical men are miles away, to supplement which lack of helpers she considered the village nurse existed. She urged the establishment of County Associations, and the necessity for enlisting the help of County Councils on behalf of the rural district nursing system in the shape of nursing scholarships. Lady Laura considered that it was impossible in most country districts to raise the sum required for the establishment of a Queen's nurse, making it necessary "to employ a second class of nurse, namely, village nurses and certificated midwives, trained for at least six months in a district, such as those trained at Plaistow or the Clapham Maternity Hospital." Lady Laura went on to explain in detail the best way to launch a scheme of such district nursing, to form village associations and county federations. Speaking of the nurse herself, her opinion was that "the most successful village district nurses have been drawn from the domestic servant or small tradesman class, and are women of thirty to forty-five years of age."

Sister Katharine put forward once more all the arguments on behalf of the legal registration of midwives, stating her view that "no power exists to prevent a neighbourly person from acting as midwife in an emergency, and this being so, no amount of opposition can ever accomplish their extinction," the problem, therefore, being not to decide if midwives shall exist, but whether they shall be as bad or as good as present conditions allow. Sister Katharine recounted some of the current objections to the Registration Bill, stating her conviction that those members of the medical profession who opposed it did not appreciate that the trained midwife is infinitely more ready to call in the doctor than the Gamp. Going on to consider the question of the training of midwives, she greatly regretted that present conditions limit it to three months, a period upon which an increase could hardly be hoped for while midwives have to bear the expense of their own training. All should bear in mind that to make good midwives they must of necessity be skilled maternity nurses; and, in her opinion, no pupil ought to be allowed to present herself for examination by the authorities of her training school until her nursing capacities have reached as high a standard as her technical knowledge.

A most interesting discussion followed, Miss Hughes, of the Nurses' Co-operation, who made an admirable speech, so holding her audience that when the time limit expired, her remarks being unfinished, she was brought back again with great applause to conclude them. Agreeing with all that had been said on the appalling need for trained maternity and nursing aid, Miss Hughes put her finger on the weak spot in the "village" nurse scheme, the spot "where its greatest strength should lie, the nurse herself," and begged those interested in forwarding the cause of nursing to hesitate before launching half-trained women upon the country districts. Remember, said Miss Hughes, these women hold human lives in their hands, and shall we not give them time enough to be thoroughly trained? Most of them come from the very class whose superstition and ignorance they have to overcome, yet some of them with only three months' training are left to their own resources with neither sister nor doctor to guide them. She pleaded for further training for district nurses, as distinct from the midwife or monthly nurse, for whom three months' training had now to be accepted as the minimum.

Mrs. Rickman, representing the Midwives' Institute, laid special stress upon the point that no improvement could be looked for in the present state of things until legislation on the midwife question was an accomplished fact. She explained that the Bill to be brought before Parliament later on would be called a "Licensing" instead of a Registration Bill, the difference consisting mainly in the fact that the midwives would be under local authority instead of a London Registration Board.

Miss Haycroft, Miss Bromley, Mrs. Rogers, the Countess of Leicester, Miss Wright, L.O.S., Mrs. Gurney Buxton, and other ladies with special knowledge on the subject also spoke, and a strong feeling was evidently aroused in favour of legislation on this important matter.

### The International Conference of Women Workers.

### A WORD OF WARNING.

At the annual meeting of the council of the N.U.W.W., held on the third day of the recent conference at Norwich, the arrangements for the International Congress which is to be held next June in London came up for consideration. The programme of subjects for discussion at the Congress and the names of proposed speakers in the various sections were read, and it was requested that further lists of names might be sent in, if there were any to be suggested. Now under the heading "Professional" comes "Nursing," with three sub-sections: (*a*) Professional Training and Status of Nurses for the Sick; (*b*) Work of the Professor in Nursing; (*c*) Nursing Organisations. It is on the question of speakers on these subjects that a word of warning must be addressed to the members of the English Committee of Arrangements. The list of proposed speakers (a very short one) on this important branch of women's work is absolutely inadequate. Nursing is entirely unrepresented by those prominent women whose names are chiefly associated with the recent advance of trained nursing in this country. Where are the matrons of our great London hospitals? Not one was mentioned in the list of proposed speakers. If women like Miss Gordon, of St. Thomas's, Miss Lückes, of the London, Miss Monk, of King's, Miss Thorold, of Middlesex, Miss Nott-Bower, of Guy's, Miss Gordon of Charing Cross, Miss Wedgwood, of the Royal Free, who have been long guiding our greatest training schools, are too occupied with their heavy work and responsibilities to find time for public matters, surely there are and must be other truly representative women who will be prepared to sacrifice something to speak for their profession at so important a meeting as this congress of women cannot fail to be if rightly managed? It would be better to leave nursing out of the programme altogether if it is not to be represented by those most interested in its development and best qualified to speak on the subject. Let us urge upon the Committee of Arrangements that they leave no stone unturned to get this important section rightly and adequately represented. It is not too much to say that those nurses present at the Conference were dismayed at the entire absence of the best known names in the nursing world from the list of speakers put before them. And let us beg those matrons who receive a request from the Committee of Arrangements to speak at the meetings next June to think more than twice before they decline to do so. By the world—for women are coming from all parts of it, from the Colonies, from Europe, from the United States—even the best motives for remaining in the background will hardly be recognised. It will only appear that nursing as a calling for women in England can only find one or two by no means representative nurses to speak on its behalf. Seeing that the trained nurse is perhaps the most highly specialised product of the new position occupied by women at this end of the nineteenth century, it will surely be strange if some of the most prominent members of this great body in England do not come forward at the Congress of 1899.

### Hospital Amenities at Norwich.

A Special Correspondent of "The Hospital" writes: Probably all readers of the articles entitled, "Institutional Amenities," which are appearing in another part of The Hospital, will agree in the opinion that if matters are really as there represented, it is a fact to be deeply deplored by all lovers and upholders of our voluntary hospital system. But are the "amenities" really so lacking, and is it a fact that "hospital" in England has become a synonym not for "hospitality" but for brusque discourtesy? My experience is a far different one. It has been my fate to enter the doors of most, if not all, of the London hospitals in turn, as a Press representative. I can hardly recall one instance where the reception has not been courteous; frequently a first visit has resulted in many another, and I could give a respectable list of institutions where a welcome greets one at any hour, and where hospitality is meted out to visitors with no sparing hand. Let me take one instance. I have just returned from representing The Hospital at the Women Workers' Conference at Norwich, during which I have been a guest at the Norfolk and Norwich Hospital, the recipient of a most kindly welcome, and every possible courtesy and attention. Visits to other provincial hospitals are amongst my pleasantest experiences. And so it should be, and, so far as at least one other individual opinion goes, is at the great majority of institutions. It would be well if such opportunities as congresses and large gatherings in various centres were more generally seized by hospital authorities for proffering their hospitality. To be, even for a short time, under the roof of one of our splendid charities, and a witness of its innermost economy, is the best possible way of learning to appreciate its work. Such hospitality does good all round; it most certainly "blesses him that gives and him that takes"; the institution benefits by the greater knowledge gained of its methods and labours, and surely only the most blunted intelligence can enter the walls of one of our great hospitals without falling under the fascination of its noble work and aims, and profiting by its educative influence. The best answer to a charge of discourtesy will be to throw even more widely open the doors of our hospitals, so that they may become yet larger "centres of civilisation," and extend their influence over an ever-increasing circle of personal acquaintances and friends.

### The Royal National Pension Fund for Nurses.

Few matters have occasioned more interest among nurses lately than the recently announced result of the quinquennial valuation of this Fund. All the members of the Fund are well aware that a large sum of money has been invested from time to time by some of the merchant princes of London to add to the investments of the nurses, and that the accumulated interest on this Fund is divided among policyholders every five years. This Bonus Fund now amounts to the large sum of over £70,000, and it is so constituted that it shall always go on increasing, so that whatever number of nurses may join the Pension Fund it shall not fall short of its purpose. The interest on this Fund is allowed to accumulate for five years; one quarter of the total amount thus realised is then added to the capital, and the remaining three quraters are placed to the credit of the policy holders. The last occasion on which the interest of this Bonus Fund was divided was in 1892; the interest then amounted to £4,302, and so three-fourths of this, or £3,327, went to increase the policies held by the nurses. The Donation Bonus Fund since then has been largely added to, not only by the accumulation of interest, but by further donations, and this year the amount of interest to be divided is more than doubled. The total is £8,864, and three-quarters of this, amounting to £6,648, has been divided among all the nurses holding policies on December 31st, 1897. The remaining quarter goes back, as we have explained, to increase the capital, and though it is rather too much to expect that the interest on this fund should be found to be doubled every five years, yet there is every security that each valuation will find it very materially increased.

But it must be borne in mind that though this Bonus Fund is already wealthy and is steadily increasing in size, the number of policies is very considerable also. There are now very nearly five thousand members of the Pension Fund, and, supposing that all the members received an average sum, the bonus from this source would amount to little more than a guinea apiece. The awards of the Bonus Fund are not, however, settled on this easy method of average. The bonus additions cannot be regarded as in the nature of a charitable distribution; rather, it may be said that the Denation Bonus Fund is a machinery for procuring those who take out policies better interest on their money than they could get elsewhere, and the awards are therefore calculated on the basis of "Whosoever hath to him shall be given." Mr. George King, the distinguished actuary entrusted with the delicate task of assigning to each policyholder her just proportion of this added interest, has calculated with infinite pains exactly what each policy will be worth in money value when its holder enters upon her annuity. In order to do this he has reckoned up how much each one has paid so far, and the exact amount, including interest, which they will have paid at the rate of their present premiums before their task of saving is completed, and they enter upon the fruits of their labour. Having made this calculation for every single policyholder, he then proceeded to divide the £6,648 among them so that each should receive a proper proportion of interest on the lump sum which they are aiming at. It will be seen then that the amount of bonus will vary greatly between that assigned to the nurse who merely, as it were, plays at saving for an annuity of some £4 or £5 a year, and that received by those practical women who realise that under £30 or £40 a year they can scarcely expect to pass their days in even tolerable comfort when they retire from work. If the latter receive eight or ten times the amount of bonus assigned to the former, it is because these bonus additions were subscribed not to make up pensions for those who through misfortunes of various kinds are unable to make proper provision for themselves—this is rather the province of the Junius S. Morgan Benevolent Fund—but to increase the rate of interest on the savings of those who can and do put by effectually for the future.

We have shown that the amount added to the policy of each individual nurse from the Bonus Fund will vary considerably, and for those who contribute little must inevitably be very small. But there are two points to be remembered. This amount, small though it be, is added every successive period of five years, and, judging by the rate of difference between this and the former distribution, it will tend to increase rather than diminish, even allowing for a large increase in the number of those who will participate in it. It will, moreover, continue to be added even after the poli cy holder enters on her annuity, so that a nurse entering the Fund, say, at thirty, will receive in the natural course before, in these days of vigorous age, she begins to feel infirm, eight or ten of these pleasant little bonuses, and thus the result is vastly more important than appears at first sight.

So much for the Donation Bonuses, which form a special feature of the Royal National Pension Fund for Nurses.

There is, however a new factor at the quinquennial valuation this year, wholly absent five years ago, but likely to present itself in even more imposing figures as time goes on. This is the sum of £10,303, which represents the profits made during this period, now available, according to the constitution of the Fund, to distribute among the policyholders. Five years ago the Fund was in its infancy, having had many costly initial expenses to meet, and the amount of profit realised amounted to little over £700. It is now fairly launched as a first-class Life Insurance Association, and its profit proves to be larger than even its most sanguine friends had ventured to predict. It is necessary to the right understanding of this matter to explain in what way these profits have been secured.

All Life Assurance Companies of the first order, that is, all those which are absolutely secure, base their rates of insurance on very careful calculations as to the probable duration of life, compiled from statistics which are being continually revised according to the altered conditions of every-day life. It will be found that their rates are all very much alike, though they compete with each other by offering various conveniences to their clients. The limits of variation in the cost of a policy at all these first-class offices are very confined, though one office may make rather better terms than another for some special class of "lives." There is very little difference then in the amount of profit which accrues from the actual gain on policies in these various offices; that source of profit is reduced by competition almost to a vanishing point. The real profit is made on the disposition of the funds which are entrusted to the management for investment. It is, as it were, a big banking concern. You trust them with your money, and they do the best they can for it.

In ordinary insurance offices the profits of this banking business are divided among the shareholders who have started it with their capital, and if the profits will allow of it, something is set aside occasionally as a bonus to the policy holders. The Pension Fund is in the unique position of having no shareholders, and so every penny of the profit made by wise investment of the nurses' savings is allowed to accumulate, and is added every five years to the Donation Bonus, of which we have already spoken.

There is no need to say anything more of the principles on which this Profit Bonus is divided. They are exactly similar to those which have been fully explained above. But it is worth noting that the Profit Bonus, which has now been declared, amounts to nearly double the sum available for distribution out of the Donation Bonus, and that, judging according to the nature of business enterprises where "nothing succeeds like success," there is solid ground for believing this will be far exceeded when the next quinquennial valuation comes round in due course. To those who have felt a little disappointment perhaps that £70,000 should not produce for each of 5,000 policyholders a really comfortable nest-egg of interest, this division of profit comes as a very timely consolation and encouragement. It shows them that they are shareholders in a really prosperous business, and should—if they are still inclined to wish their share of interest had been greater—encourage them to use the Fund courageously as the best investment for their savings, instead of putting in only just enough to secure a bare pittance later on, and placing the rest of their money in gimcrack securities, vouched for on the faith of their particular friends.

One word more. The profits of every concern of this kind depend not only on the business ability displayed in investing the money, but on the economy of its management. It has become the custom to test both charitable and business enterprises by reckoning how much per cent. of the income is spent on the management. In glancing down the figures of one large society after another, it is impossible not to be struck with the large proportion of the income which is almost invariably swallowed up in the expenses of the offices, the advertising, the salaried officials, and the printing. It is not uncommon to see that £1 out of every £8 or £10 received is absorbed in this way, and if the Society has kept its management expenses down to £1 out of every £15 it may be called economical. Hence it is a striking fact that the Royal National Pension Fund for Nurses, in spite of its intricate financial relations with its policyholders (differing in each individual case), has succeeded in conducting all its business at a charge on the total income of no more than 4 per cent., or in other words expends but £1 in every £25 on management. All who have experienced the unvarying courtesy and ready attention accorded to policyholders whenever—seeking solution of some knotty point—they make a call at headquarters, have reason to admire this economical result, the fruit of no short-sighted policy of curt answers and officialism, but of strict business ability, conspicuous above all in the rare art of never seeming to be in a hurry.

### "Feed My Lambs."

"Our Father," a humble, sweet voice said,
"Give us this day our daily bread."
And the sad little prayer, with its plaintive tone,
Went winging its way to the great white throne.
But no answer came, and the voice grew faint,
As it trustfully murmured its childish plaint,
"Our Father, oh hear us as we pray!
"Give us our daily bread to-day."
Still silence reigned, and the dim blue eyes
Were raised to heaven with a vague surprise.
"Our Father!" again the white lips said,
"Give us this day our daily bread."
"Our Father!" and even then there fell
A shadow deeper than tongue can tell.
And into the darkness a spirit fled,
And the cry was ended for daily bread.

The clashing bells were the call to pray;
And the city churches were full that day.
The knees of the rich were bent in prayer,
The incense rose on the hallowed air,
Tunefully thousands of voices said,
"Give us this day our daily bread."
And away in the home of the quiet dead,
The recording angel bowed his head.

### Queen Victoria's Jubilee Institute for Nurses.

(Scottish Branch.)

We have just received the quarterly report of the Scottish Council, and learn that there are at the present time in affiliation with the Queen Victoria Jubilee Nurses' Institute 94 Scottish branches. These include three County Associations, and the nurses number 164.

In consequence of the increased number of branches and of probationers in the Training Home, the Scottish Council have found it necessary to increase their staff, and Miss Cowper has been appointed assistant to Miss Wade, the inspector, and Miss Myers second assistant in the Home.

During the three months 10 nurses completed their training, and received appointments to Airdrie (second nurse), Jedburgh, Port Glasgow (second nurse), Rothesay, Lochwinnoch, Hamilton (third nurse), Innerleithen, Loanhead, Killean and Wick. Queen's nurses at the following places have been inspected and reported on to the Executive Committee: Shielbridge Kilchoan, Tiree, Iona and Bunessan, Oban, Easdale, Ardrishaig, Inverary, Strachur, Islay, Tarbert, Campbeltown, Wemyss, Greenock and Lerwick. The Scottish Council are directly responsible for five Queen's nurses, 24 Queen's probationers in various hospitals for training, and 18 district probationers. Dr. Harvey Littlejohn and Dr. Milne Murray have completed their courses of lectures on hygiene and on obstetrics, and 26 district probationers were examined. Increased facilities for teaching cookery have been arranged for the probationers in the Training Home, and the work progresses satisfactorily,

### Appointments.

National Hospital for the Paralysed and Epileptic, Queen Square.—Owing to ill-health, Miss Morris, the matron of the convalescent home, is about to retire, after a service of nearly 28 years as nurse, sister, and matron, of which nine have been spent in the last capacity in the old and new home at East Finchley. Miss Morris has gained the esteem and regard of all with whom she has come into contact, and the hospital authorities have marked their appreciation of her services by the grant of a pension. Miss Kate Headford, now night sister of the hospital, will succeed Miss Morris.

Union Infirmary, Stafford.—Miss Mina Morton has been appointed Superintendent Nurse of the above. She was trained at the Fir Vale Infirmary, Sheffield, and her latest post was that of charge nurse at the Fever Hospital, Stockton-on-Tees. Miss Morton holds a certificate of midwifery from Queen Charlotte's Hospital, and has been district nurse at Kilburn, Norwich, and Oswestry.

Royal Victoria and Albert Docks Hospital.—Miss C. Graham Knight, formerly sister of the "Northumberland" Floor at the Dreadnought Hospital, Greenwich, has been appointed Matron of their Branch Hospital in the Royal Victoria and Albert Docks. It is at this hospital that the new School of Tropical Medicine is being established. Miss Knight received her training at the London Hospital.

Dundee Royal Asylum.—Miss Annabel L. Irvine has been appointed Matron of this institution. Miss Irvine was trained at the Glasgow Western Infirmary, where she afterwards became sister, and subsequently acted as staff nurse at the Perth District Asylum, Murthly.

### Wants and Workers.

The Matron, the Accident Hospital, Mansfield, would be glad of a Bath chair for the use of the patients

### A Book and its Story.

### WITH KITCHENER TO KHARTOUM.

Perhaps at this moment there are no two words whose sound stirs so deeply and so insistently the heart of the British nation as the two names that give its title to the book* whose story we have selected for review to-day. Mr. G. W. Steevens, in his romance of the Sudan campaign under the fitting heading, "With Kitchener to Khartoum," sets forth with all the brilliant versatility of which he is so distinguished a master the history of the failures and achievements which culminated in the triumphal entry of our troops into Khartoum.

"The curtain comes down; the tragedy of the Sudan is played out. . . . It has cost us much, and has profited us—how little? It would be hard to count the money, impossible to measure the blood. Blood goes by quality as well as by quantity. . . . By shot and steel, by sunstroke and pestilence, by sheer wear of work, the Sudan has eaten up our best by hundreds. . . . And what have we to show in return? At first you think we have nothing; then you think again, and see we have very much. We have gained precious national self-respect, and by the vindication of that self-respect the great treasure we won at Khartoum is worth the price we paid for it. . . . We gave the lives of men we loved, and we conquered the Sudan again. Now we can permit ourselves to think of it in peace. We undertook to leave Egypt, but if we undertook to evacuate the old Egypt we have fathered a new one, saved from imminent extinction by our gold and our sword. Without us there would have been no Egypt of to-day, and," concludes the author with significance, "what we made we shall keep." And what about the men, the brave, distinguished soldiers, with the man at their head, who have brought the contest to so swift and sure a close? First of all there stands out the leader, known to everyone as "the Sirdar," which title he assumed in succession to Sir Francis Grenfell in 1894. Nor was he slow to show that he meant to be "Sirdar" in name as well as in fact, for it has passed into history that under his *régime* the weak and restive Khedive had to do public penance. Here stands the hero. He is "forty-eight years old by the book," one of the original 25 officers told off for the new Egyptian army, of which he was appointed commandant in 1882, and "in Egypt and the Sudan he has been ever since," on the staff generally, in the field constantly, alone with natives often, mastering the problem of the Sudan always. The right man in the right place, undoubtedly, but not unaware at any time of the debt due from himself to the failures, as well as achievements, of those who had preceded him. "For Anglo-Egypt he is the Madhi," the man who sifted experience and corrected error; who has worked at small things and waited for great; marble to sit still and fire to smite. This is the man, then, who "has made himself a machine to retake Khartoum." In appearance "he stands over six feet, straight as a lance, and looks out imperiously above most men's heads. . . . Steady, passionless eyes, shaded by decisive brows . . . a long moustache, beneath which you divine an immovable mouth; his face is harsh, and neither appeals for affection nor stirs dislike. . . . Other generals have been better loved; and, high tribute, none was ever better trusted. His officers are wheels in his machine." They are worked as mercilessly as he works himself. "Marriage interferes with work," so he will have no married officers in his army. "If you suppose, therefore, that the Sirdar is unpopular, he is not. No general is unpopular who always beats the enemy." When columns march out into the dark night at his command and "fight till the dawn with an enemy they have never seen, every man goes forth with a tranquil mind. He may come back personally and he may not; but you may bet your boots the Sirdar knows—he would not fight if he weren't going to win. The brain and the will are the essence and the whole man. He has no age but the prime of life; no body but one to carry his mind; no face but one to keep his brain behind; and the brain and the will are so perfect in their workings that the Sirdar is never in a hurry. Whilst others reflect he is acting. A desert route had been suggested connecting Kirosko to Halfa; but the Sirdar used it. Desert railways had been projected; the Sirdar made one. That summarised in one instance is the working of the Sudan Machine."

One word as to the man who is the "sword arm" of the Egyptian Army—General Hunter. "In all he does he is the true knight-errant—a paladin drifted into his wrong century. For fourteen years he has been in the front of all the fighting on the southern border. When there was fighting he always led the way with his blacks, whom he loves like children, and who love him like a father. A cheery, lovable personality surely. One of those happy men whom Nature has made all in a piece—consistent, simple, unvarying; with keen, conquering, hazel eye, looking outward and upward like an eagle's, having the youthful vivacity of a schoolboy."

Of the battles of the Atbara and Omdurman there are vivid moving descriptions, and the author makes the following "unprofessional" criticisms upon them: "If the unprofessional observer may allow himself the liberty the battle of Omdurman was a less brilliant affair than the Atbara; on the other hand it was more complex, more like a modern battle. The Atbara took more fighting, Omdurman more generalship. Success in each case was complete and crushing."

The following extract illustrates the cosmopolitan composition of the Sirdar's force as, after a month's menacing and evasion, it was at last brought face to face with the enemy at the battle of the Atbara. . . . "The word came, and the men sprang up—all England and all Egypt, and the flower of the black lands beyond Birmingham and the West Highlands, and half-regenerated children of the earth's earliest civilisation, and grinning savages from the utmost swamps of Equatoria, muscle and machinery, lord and larrikin, Baliol and the Board school, the Sirdar's brain and the camel's back—all welded into one, the awful war machine went forward into action . . . and the battle of the Atbara has definitely placed the blacks—yes, the once-contemned Egyptians—in the ranks of the very best troops in the world."

In conclusion, we recommend our readers to secure without delay the book for themselves that they may follow the fortunes of the Sirdar and his army for themselves.

* "With Kitchener to Khartoum." By G. W. Stevens. (Publishers: Blackwood, Edinburgh and London. 6s.)

### Where to Go.

Nursing Lectures.—On Wednesday afternoons, at a quarter past four p.m., Mrs. Clare Goslett, a member of the Sanitary Institute and one of the lecturers to the Queen's nurses, will give a course of four lectures on preventable disease. Tickets may be obtained from Miss Strong at 6, Upper Baker Street, where the lectures will be held.

Parke's Museum, Margaret Street, W.—Under the auspices of the Childhood Society an important series of lectures is being given on Thursday evenings at eight p.m. On November 3rd Sister Grace reads a paper on "The Educative Uses of Organised Play," and on the 10th inst. Mr. H. Hollman's subject will be "Discussion."

### Everybody's Opinion.

**[Correspondence on all subjects is invited, but we cannot in any way be responsible for the opinions expressed by our correspondents. No communication can be entertained if the name and address of the correspondent is not given, as a guarantee of good faith but not necessarily for publication, or unless one side of the paper only is written on.]**

### THE POOR LAW NURSE.

"A WORKHOUSE NURSE" writes: It was with very keen interest I read the article under the above heading in THE HOSPITAL, for after seven years' conscientious service in provincial workhouse infirmaries I can fully endorse the statements made by your correspondent. The public know little or nothing of the many difficulties and tyrannical annoyances which fall to the lot of the workhouse nurse, and in the interest of both guardians and nurses the whole thing wants threshing out. The system under present regulations is rotten at the core, and there is no denying the fact that the master and matron are the cause of most of the friction due largely to the fact that in the majority of cases they are untrained, and often uneducated, to fit them for their responsible position, and under whom the trained nurse is placed. The master and matron rule supreme. The law gives them so much power, and they can manage to hoodwink guardians whenever necessary. It would take too much of your valuable space to attempt details. Sufficient to say the trained nurses are looked upon by masters and matrons as beings created to annoy instead of to help them. I know of several instances where the master and matron have succeeded in inducing guardians to appoint untrained women in preference to trained nurses. To a woman who knows her work the reason is obvious. To quote a master's own words only a few weeks back at a Poor Law conference, he said these trained "nusses" come with ideas of their own, and since we have ceased to have "trained nusses" all goes smooth. But, alas for the poor patients who need skilled nursing; and yet these "so-called nurses" are paid and placed on the same standard as a nurse who has given time and money, attended with close study, to enable her to qualify for her profession. Is it possible that this state of affairs can exist without friction? As regards complaints of inmates against the nurses, I have known of patients being bribed and encouraged to rebel and complain against the nurse, providing she refuses to play into the hands of the master. It is not difficult to encourage complaints, considering the class of people we get in our workhouses. They require most careful tact. It is all gain for them to complain; they have nothing to lose—nothing at stake, and so the workhouse nurse goes drudging along, often badly housed and badly fed. She must never be tired, and must never need recreation; the consequence is she either throws up the work in disgust, or falls into a dead, dull routine with warped energies. Another important item is the issuing of stores, and the difficulty experienced in getting a sufficient supply of bed and body linen. The house authorities have full run of the stores for whatever is needed, but those of the infirmary must ask for every trifle, and that trifle they only get after a struggle. I have never yet known what it was to get supplies without grumbling and insulting remarks from the matron. The able-bodied are considerably better clothed than the infirmary patients. Fortunately, the diets are left to the medical officer, but very often the doctor is led by the master. There are "wheels within wheels," and when (as is often the case) the doctor plays into the hands of the master the workhouse nurse is to be pitied. I may say I have been exceedingly fortunate as regards working under good medical officers, who have always maintained fair play for the hospital. I am considered a born nurse and organiser, and regret that I feel compelled through over-strain, brought about chiefly by petty friction, to give up a work I am devoted to. I would like to give some of my experiences, but I fear I have already trespassed on your valuable time. Reform can only be brought about by separating house from infirmary; let the nurse be responsible to her superintendent and medical officer. Until this is done no satisfactory results can be attained. The workhouse nurse will hail the day when Poor Law regulations are reorganised, and the Local Government Board come to the rescue.

### Notes and Queries.

**The contents of the Editor's Letter-box have now reached such unwieldy proportions that it has become necessary to establish a hard and fast rule regarding Answers to Correspondents. In future, all questions requiring replies will continue to be answered in this column without any fee. If an answer is required by letter, a fee of half-a-crown must be enclosed with the note containing the enquiry. We are always pleased to help our numerous correspondents to the fullest extent, and we can trust them to sympathise in the overwhelming amount of writing which makes the new rules a necessity.**

**Every communication must be accompanied by the writer's name and address, otherwise it will receive no attention.**

*Sanatoria for Consumptives.*

(58) Could you kindly say if Dr. Broadbent has started a sanatoria for consumptive patients of the middle class?—*Nurse Edyth.*

We presume that Nurse Edyth refers to the proposals which were lately made at a meeting held under the presidency of Sir William Broadbent to promote the organisation of a national crusade against tuberculosis. We are not aware, however, that the proposal has yet taken a concrete form.

*French Medical Dictionary.*

(59) Kindly give me the correct title and name of publishers of the French Medical Dictionary referred to in THE HOSPITAL some weeks ago.—*C. G.*

Possibly "Lexique Formulaire des Nouveautés Médicales," by Professor Paul Lefert, published by J. B. Baillière et Fils, Paris, price 3 frs., which was reviewed some time ago in THE HOSPITAL.

*Fire-Proofing Dresses.*

(60) I shall be much obliged if you will kindly tell me how to make cotton dresses fireproof?

It cannot be successfully done by an amateur.

*Eczema.*

(61) Nurse E. would be grateful to any fellow nurse who could recommend a remedy for eczema affecting the face only. Lanoline, zinc, vaseline have all been tried without any result.

Eczema is a baffling and troublesome disorder that requires more than local treatment. We should recommend Nurse E. to consult a medical practitioner.

*Sanitary Inspector.*

(62) Can you inform me how to become a sanitary inspector, and to whom should I refer to for instructions?—*Nurse J.*

Apply to the Sanitary Institute, Margaret Street, W.

*Massage.*

(63) I should be glad to know if a trained nurse could make a living by massage and daily nursing, and the best locality near London in which to start?—*Subscriber.*

It is all a question of connexion, therefore the locality must be one in which a nurse is well known. We refer our correspondent to the Society of Trained Masseuses, 12, Buckingham Street, Strand.

*Egypt and Italy.*

(64) Can you give me the address of any English nursing homes in Egypt or Italy?—*Nurse Edith.*

Apply, The Committee, Victoria Nursing Institute, Alexandria; or Miss James, the English Nursing Home, Cairo; or Miss Violetta Fasulo, No. 7, Via Rondinelli, Mezzanino, Florence.

*Quotation.*

(65) Do you think the writer of "Commit thy words unto Jehovah," in "Reading for the Sick," would kindly tell me where the words are to be found?—*Busy.*

"Commit thy words unto Jehovah" is a quotation by Miss F. R. Havergal in "Royal Commandments," in a chapter headed by the text, "Commit thy works unto the Lord."

*Indian Nursing Service.*

(66) 1. Kindly tell me does one require much interest to get into the Indian Nursing Service? 2. If appointed, to what part of India is one likely to be sent?—*T. N.*

1. Candidates for the Indian Nursing Service must be ladies by birth and education, and hold a three-year certificate for training in general nursing. Preference is given to the widows and daughters of army officers. Apply to the Army Medical Department, 18, Victoria Street, S.W. 2. It is impossible to guess.

*Coley's Fluid.*

(67) 1. Where can I get Coley's fluid? 2. Is it expensive? 3. Can it be used with a view to disperse fibroid tumours?—*E. F. A.*

"E. F. A." is evidently under a complete misconception if she thinks that Coley's fluid is a medicine to be "dispensed." The administration of the toxins which go by the name of Coley's fluid is a matter of considerable gravity. It is an exclusively medical affair, and should not be undertaken even by a medical man until he has made himself fully conversant with the consequences which may ensue, and then only after the patient has had the matter carefully explained to him.

*Polished Floors.*

(68) I am having the floors of two wards "paraffined," as mentioned in Sir Henry Burdett's classical work. Can you or any of your readers tell me the best way to treat the surface afterwards?—*Hospital Surgeon.*

Polish either with beeswax and turpentine or "Ronuk." Some matrons prefer the one, some the other.

*Uniform Dolls.*

(69) "E. P." would be glad to know if the uniform dolls are still in existence, and, if so, if they would be available to exhibit at a bazaar to be held, in December.

The uniform dolls are not now in existence.

